# Impact of different work organizational models on gender differences in exposure to psychosocial and ergonomic hazards at work and in mental and physical health

**DOI:** 10.1007/s00420-021-01720-z

**Published:** 2021-05-29

**Authors:** Maria Cristina Migliore, Fulvio Ricceri, Fulvio Lazzarato, Angelo d’Errico

**Affiliations:** 1 Institute of Economic and Social Research of Piedmont, Turin, Italy; 2 Department of Clinical and Biological Sciences, University of Turin, Regione Gonzole 10, 10043 Orbassano, Turin Italy; 3 Epidemiology Unit, Piedmont Region, ASL TO3, Grugliasco, Turin Italy; 4 Department of Medical Sciences, University of Turin, Turin, Italy

**Keywords:** Work organization, Gender, Work hazards, Health, Epidemiology

## Abstract

**Purpose:**

To examine differences between genders in exposure to psychosocial and ergonomic factors at work and in work-related health, according to different work organization models.

**Methods:**

The study population included a sample of 9749 (women: 37.1%) and 10,374 (women: 39.9%) employees who participated in the 2010 and 2015 European Working Conditions Surveys, respectively. Multiple Correspondence Analysis was applied to work characteristics reported by workers to estimate principal components, followed by Hierarchical Clustering on principal components to identify clusters of work organization models. Gender differences in exposure to work hazards and health outcomes were assessed through Poisson robust regression. Differences of PRs across organizational models were tested through interaction between gender and type of work organization.

**Results:**

Three organizational models were identified in 2010, including lean production, Tayloristic production, and a “reflexive production” model, whereas in 2015, a “simple” or traditional model was also found. In 2010, women employed in companies adopting the Tayloristic or the lean production models were more likely than men to be exposed to unfavourable psychosocial and physical work factors, and to report musculoskeletal pain, compared to those belonging to reflexive production. In 2015, a significantly higher female/male ratio persisted in lean production for exposure to high job strain and for carrying/moving heavy loads, whereas gender differences in Tayloristic and traditional production were quite similar to those of reflexive production.

**Conclusions:**

Our results suggest that employment in workplaces characterized by lower monotony, repetitiveness, and production constraints may contribute to reduce exposure to job strain among working women.

**Supplementary Information:**

The online version contains supplementary material available at 10.1007/s00420-021-01720-z.

## Introduction

Although the relationship between quality of work and health has been investigated for several decades, the issue of women’s well-being in the workplace is still an under-researched area (Connerley and Wu 2016). Female work, especially manual one, is often characterized by monotonous, repetitive actions, with static effort and multiple simultaneous responsibilities, which potentially threaten both women’s physical and mental health; work areas, tools, and pace derive from a work organization and an equipment endowment created for a male population and may not be suitable for women, who have a different anthropometric structure (Bond et al. 2004; Messing 1999, 2017).

Different epidemiological studies have found women at higher risk of developing some work-related diseases, especially musculoskeletal (de Zwart et al. 2001; Hagberg and Wegman 1987; Park et al. 2017; Roquelaure et al. 2006) and mental disorders (Beauregard et al. 2018; Bilodeau et al. 2020; Blehar 2006; Kuehner 2003; Roxburgh 1996; Wege et al. 2018). Although the mechanisms underlying such excess risks are still unclear, it has been proposed that they would stem, at least in part, from differential exposure to physical and psychosocial factors at work between men and women (Krieger 2003; Quinn and Smith 2018).

In general, from the literature, women appear more exposed than men to the hazards currently most diffuse among workers, i.e., ergonomic and psychosocial ones, including awkward and tiring postures (Eng et al. 2011; Hooftman et al. 2005; Nordander et al. 1999), hand/arm repetition (Sterud 2014), standing and bending (Sterud 2014), walking long time (Bauer et al. 2009), low job control (d’Errico et al. 2011; Hooftman et al. 2005; Josephson et al. 1999; Messing et al. 2009), high demand (Sterud 2014), high job strain (d’Errico et al. 2011; Ibrahim et al. 2001; Karlqvist et al. 2002), repetitive work (Eng et al. 2011; Messing et al. 2009; Nordander et al. 1999; Strazdins and Bammer 2004), effort–reward imbalance (Johannessen and Sterud 2017; Sterud 2014), and sexual harassment (Das 2009; Messing et al. 2009; Sterud 2014).

However, the results on exposure to adverse work factors appear inconsistent among studies, as shown by a systematic review on the subject, which found a higher prevalence of exposure in women only for job insecurity, low job control, and worse contractual working conditions (Campos-Serna et al. 2013).

The higher exposure of women to workplace ergonomic and psychosocial factors has been interpreted mainly as attributable to women’s work segregation, i.e., the selective employment of women in certain economic sectors and in lower status jobs, in terms of professional position and responsibilities. Two types of work segregation are commonly distinguished: horizontal and vertical. The horizontal segregation means that women are generally employed in sectors and jobs different from those of men. The economic sectors with the highest concentration of female workers are health care, trading, education, tourism, services, and domestic work, whereas men are mainly employed in construction, manufacturing, transportation, agriculture, and finance. Few sectors can be actually defined as mixed, such as the public sector and some branches of manufacturing, like textiles and garment or food production, with women more often employed in small companies. The vertical segregation means that women are less represented in executive or more remunerated positions, with hierarchies at work clearly dominated by men, who generally have higher wages, more secure jobs, and more career perspectives (Blau and Kahn 2007; Fagan and Burchell 2002). Vertical segregation is mainly driven by two mechanisms which limit the possibility for women to access higher positions. The first one has been named “glass ceilings”, intending that for female workers to be promoted at top level positions is very difficult because of invisible barriers created by men’s power and attitudes. The second is that of “sticky floors”, meaning that women have more difficulties than men in leaving entry-level job for higher positions (Pyle and Bond 1997). In this framework, it is pointed out that even if they are employed within the same job title, women and men usually perform different tasks and activities (Messing and Mager Stellman 2006), which may expose them to different types of occupational hazards, or to the same ones, but with different exposure intensity and frequency (Messing et al. 1994).

However, the gender segregation approach tends to emphasize the issue of hierarchy, power, and position in it, neglecting other organizational aspects relevant to the gender perspective (Quinn and Smith 2018), such as behavioural expectations concerning languages, emotions, competition, performance, collaboration, assertiveness, rationality, control, and autonomy. Other perspectives deal with these aspects and argue that organizations are gendered, as they have been “designed by men for men”, that is, they are largely defined by male practices (Acker 2012; Benschop and Verloo 2016; Burke 2014; Kelan 2018; Lewis et al. 2017; Rumens 2017; Wahl 2014). Even when managers are women, organizations tend to remain gendered in favour of men’s practices. This happens, because the managerial practices are powerfully and historically associated with a masculinist way of being and behaving, and all managers are required to conform to them (Whitehead 2014). This mechanism can explain the slowing pace of change toward gender equality at work (Benschop and Van den Brink 2014).

In this context, besides gender segregation, also employment in firms with different models of work organization may influence women’s well-being and exposure to workplace hazards, compared to their male counterparts. Even if gender equality is far to be achieved, the different types of work organization can create different social and cultural environments, and therefore bring about different well-being conditions for women (and men).

The present study aims at contributing to the debate on the role of different types of work organization models as determinants of exposure to physical and psychosocial factors in the workplace, and of workers’ health and well-being in the European Union, with a strong focus on gender differences. More specifically, objective of the study is to assess whether different models of work organization display gender differences in self-reported exposure to psychosocial and ergonomic factors at work, and in self-reported mental and musculoskeletal health. Our research builds on the work by Lorenz and Valeyre (2005), who used several work characteristics collected in the European Working Conditions Survey (EWCS) 2000 to identify clusters of workers employed in work organizations belonging to the different models. The advantage of adopting an approach based on work organization models rather than on gender-based segregation to assess gender differences in workplaces, is that: (1) is closer to industrial and economic discourses oriented to enhancement of productivity in different types of work organization; and (2) there are growing doubts that desegregation could always benefit women (Messing 2017).

Another aim of the study was to assess whether among European workers gender differences in exposure to work factors and in work-related health changed between 2010 and 2015, also using EWCS data.

## Materials and methods

### Study population

Data derived from the Fifth and Sixth waves of the Eurofound survey, undertaken in 2010 and 2015, respectively. The European Working Conditions Survey (EWCS) has been conducted every 5 years in the European countries since 1990 by the European Foundation for the Improvement of Living and Working Conditions (Eurofound). Main objective of this survey is to measure aspects of working conditions and to monitor their trend in time in European countries. The Fifth edition covered the 27 EU member countries, as well as four candidate countries (Croatia, Macedonia, Montenegro, and Turkey), two potential candidates (Albania and Kosovo), and one country as a member of the European Free Trade Association (Norway). The Sixth edition included the same countries, plus Switzerland and Serbia. The population surveyed was a representative sample of the employed population aged 15 years and over in each country (16 years and over in Spain, UK, and Norway), selected according to a multistage, stratified random sampling design, with a participation rate of 44% in 2010 and 43% in 2015 (Eurofound 2013, 2016). The final sample included 43,816 subjects (48% women) in the 2010 survey and 43,850 (49.6% women) in the 2015 survey. The interview questionnaire was composed of more than a hundred questions on socio-demographics, occupation and economic sector of employment, features of work organization and exposure to psychosocial, ergonomic and environmental hazards, as well as questions on health status, sickness absence, etc.

Analyses were restricted to employees working in the private sector in plants with at least 10 employees in five working sectors: industry; construction; wholesale, retail, food and accommodation; transportation; and financial services. After exclusion of subjects not fulfilling the inclusion criteria, 9749 workers (women: 37.1%) from the 2010 survey and 10,374 (women: 39.9%) from the 2015 survey were included in the study.

### Cluster construction

We applied the methodology proposed by Lorenz and Valeyre (2005) to characterize the work organization model of the firms where workers were employed. The authors through factor and hierarchical clustering analyses applied to data from the 2000 European Working Conditions Survey (EWCS) identified four main work organization models: traditional, Tayloristic, lean production, as well as a fourth model, denominated by the authors “learning” or “reflexive” production.

The traditional model is typical of small artisan or trade business, where work is informally organized through simple or non-codified procedures, with direct control on the workers by the employer or by the manager of the production line (Lorenz and Valeyre 2004; Mintzberg 1979).

The Tayloristic model is based on the principle of the application of scientific knowledge and engineering methods to industrial production, from which descends the subdivision of tasks into simpler actions to reduce the complexity of production processes, the strict control of the times and modes of production by the management, the separation of physical from mental work, and the careful selection and training of workers (Littler 1978).

The lean production is a model of work and labour process organization developed in the 50s by engineers of the Japanese Toyota car company, to make mass-production flexible to produce a variety of car models, improving at the same time quality and productivity. It aims at reducing all sorts of waste of time, space, and resources through a continuous improvement in the production (Womack et al. 1990). In this system, cooperation and teamwork are necessary, together with interruption of the production, to analyze in depth the reasons of a particular problem and prospect possible solutions (Delbridge 1998; Della Rocca and Fortunato 2006; Ohno 1988; Womack et al. 1990). Such workplace organization softens the Tayloristic principles of tasks fragmentation, separation of conception from execution, control, and supervision. The workers have broader skills and tasks, and the role of managers is transformed from controllers to coaches (Mathews 1989; Victor and Boynton 1998). Skilled workers with multiple tasks are required and welcomed to give feed-backs and suggestions about the design of work processes to the production engineering department (Migliore 2013).

“Reflexive production” has been proposed as another theoretical model of work organization, which was derived from the socio-technique Swedish model developed by Volvo in the Uddevalla plant in the 90s (Freyssenet 1995). This type of work organization, although also combining autonomous teamwork and quality management, differs from lean production mainly for the higher degree of responsibility placed on workers, their higher autonomy in deciding their work methods and controlling their work pace, and the high level of learning, problem-solving and task complexity requested to workers, who are also less burdened by procedural or hierarchical constraints than in the lean production (Lundvall et al. 2011).

To categorize workers’ firms into different forms of work organization, we used, as done by Lorenz and Valeyre (2005), the following 15 dichotomous variables taken from the Eurofound survey:

1. Use of team work (TEAM WORK);
2. Use of job rotation (JOB ROTATION);
3. Autonomy at work in the methods used (METHODS AUTONOMY);
4. Autonomy at work in the pace or rate at which work is carried out (PACE AUTONOMY);
5. ‘Automatic’ constraints linked to the rate at which equipment is operated or a product is displaced in the production flow (AUTOMATIC CONSTRAINTS);
6. Norm-based constraints, linked to the presence of quantitative production norms (QUANTITATIVE NORMS CONSTRAINTS);
7. ‘Hierarchical’ constraints, linked to the direct control by immediate superiors (HIERARCHICAL CONSTRAINTS);
8. ‘Horizontal’ constraints, linked to dependency of one’s work rate from the work of colleagues (HORIZONTAL CONSTRAINTS);
9. Task repetitiveness (REPETITIVENESS);
10. Perceived task monotony (MONOTONY);
11. Presence of precise quality norms (QUALITY NORMS);
12. Individual responsibility for quality control (INDIVIDUAL QUALITY ASSESSMENT);
13. Tasks complexity (COMPLEXITY);
14. Learning new things in one’s work (LEARNING NEW THINGS);
15. Problem-solving activity (PROBLEM SOLVING).

These 15 variables were used to cluster subjects, using the R package FactoMineR.

Briefly, a factor analysis for categorical variables (Multiple Correspondence Analysis—MCA) was applied to the data to identify the principal components, so that a Hierarchical Clustering on Principal Components (HCPC) could be used to infer clusters (Husson et al. 2010).

### Exposure to work hazards

We have used questions from the EWCS questionnaire on psychosocial aspects of work to build two scales of work stress, according to the two most widespread psychosocial theoretical models, i.e., the demand control, or job strain (Karasek 1979), and the effort–reward imbalance (ERI) model (Siegrist 1996). Exposure to work stress assessed through these models has been consistently associated with psychological (Theorell et al. 2015) and musculoskeletal disorders (da Costa and Vieira 2010). According to standard procedures, scores of the job strain scale were computed as the ratio of the values of psychological demand by those of the job control scale, obtained summing decision authority and skill discretion scores. The effort–reward scale was built as the ratio of demand by reward scores. Summary scores of job strain and ERI were then divided in tertiles, considering exposed to high job strain and to high ERI those subjects falling in the highest tertile of each scale. Regarding ergonomic hazards, tiring or painful postures, carrying or moving heavy loads, and repetitive hand or arm movements were considered in the study, being acknowledged risk factors for musculoskeletal disorders (National-Research-Council and Institute-of-Medicine 2001). They were assessed through single questions concerning exposure duration during the work day, considering exposed subjects reporting exposure for more than 50% of the working time. A detailed description of the construction of these indicators of exposure to psychosocial (Eurofound 2013) and ergonomic hazards (d'Errico et al. 2016) can be found in previous studies.

### Health

Regarding health, the presence of backache or muscular pain in shoulders, neck, and/or upper limbs was assessed by single yes/no questions, whereas mental health was ascertained through the WHO-5 index, a composite indicator of mental well-being elaborated by the World Health Organization (1990), considering subjects scoring less than 50 points in the index as probably affected by depressive symptoms (Blom et al. 2012).

### Statistical analysis

Prevalences of workplace characteristics, exposure to work hazards and health conditions, standardized by age class, European region, occupational social class, and economic sector were computed separately for each gender and type of work organization.

For all outcomes considered (exposure to high strain, to high effort–reward imbalance, and to ergonomic factors; low mental health and low physical health), the female vs. male prevalence ratio (PR) and the 95% confidence intervals (95% CIs) for the different types of work organization were computed using robust Poisson regression models, adjusted for age, European region (six groups: Anglo-Saxon, Continental, Eastern, Scandinavia, Southern, non-EU), occupational social class (four classes: high-skilled and low-skilled clerical, high-skilled and low-skilled manual), and economic sector (five sectors: industry; construction; wholesale, retail, food and accommodation; transportation; financial services). Prevalence ratios (PRs) from Poisson robust regression models, based on the Huber–White sandwich estimator of variance, have been demonstrated to estimate correctly relative risks in cross-sectional studies, when a high prevalence of the outcome prevents the use of logistic regression models (Barros and Hirakata 2003).

To evaluate the modifying effect of type of work organization on the relationship between gender and the health outcomes, a term for interaction was then included in the Poisson models. Interaction was considered as present if the *p* value of that term was < 0.10, given the rather low statistical power available to examine interactions in this relatively small sample.

Differences between female/male PRs of exposure to work factors and of health outcomes observed in 2010 and in 2015 in the overall sample were tested for statistical significance (*p* < 0.05) assessing heterogeneity of the PRs through random-effect meta-analysis, using the “metan” Stata command.

Finally, an analysis of gender differences by European region on EWCS 2015 data was also performed, through Poisson regression models adjusted for the same covariates as in the main analysis, testing differences among regions by heterogeneity of the PRs included in a random-effect meta-analysis.

Except for cluster construction, all other analyses were performed using STATA v. 13.

## Results

Socio-demographic characteristics of the study population in 2010 and 2015, divided by gender and type of work organization, are presented in Tables 1 and 2, respectively. The HCPC on 2010 data identified three clusters as the best number for clustering subjects, as we did not find empirical evidence for the fourth one, the traditional model. In contrast, using 2015 data, four clusters were identified, corresponding to the four organizational models, as expected.

**Table 1:** Socio-demographic characteristics of subjects included in the analyses, by gender and type of work organization

	All subjects (*N* = 9749)		Reflexive production (*N* = 3595)		Lean production (*N* = 4551)		Tayloristic production (*N* = 1562)	
	Women *N* (%)	Men *N* (%)	Women *N* (%)	Men *N* (%)	Women *N* (%)	Men *N* (%)	Women *N* (%)	Men *N* (%)
Age class								
15–24	333 (9.2)	459 (7.5)	122 (8.2)	161 (7.6)	133 (9.0)	214 (6.9)	78 (11.8)	84 (9.3)
25–34	886 (24.5)	1445 (23.7)	358 (24.2)	468 (22.1)	389 (26.5)	758 (24.6)	139 (21.1)	219 (24.3)
35–44	1042 (28.9)	1682 (27.6)	396 (26.8)	557 (26.3)	439 (29.9)	870 (28.2)	207 (31.3)	255 (28.3)
45–54	971 (26.9)	1578 (25.9)	413 (27.9)	541 (25.6)	382 (26.0)	821 (26.7)	176 (26.7)	216 (23.9)
55+	378 (10.5)	934 (15.3)	191 (12.9)	388 (18.4)	127 (8.6)	418 (13.6)	60 (9.1)	128 (14.2)
European region								
Anglo-Saxon	267 (8.4)	392 (7.5)	94 (7.0)	96 (5.3)	130 (10.1)	223 (8.4)	43 (7.6)	73 (9.6)
Continental	986 (30.9)	1900 (36.3)	434 (32.5)	649 (35.6)	395 (30.6)	958 (36.2)	157 (27.7)	293 (38.4)
Eastern	1106 (34.6)	1456 (27.8)	430 (32.2)	520 (28.5)	436 (33.8)	705 (26.6)	240 (42.3)	231 (30.3)
Scandinavia	362 (11.3)	625 (11.9)	162 (12.1)	238 (13.0)	171 (13.3)	354 (13.4)	29 (5.1)	33 (4.3)
Southern	471 (14.8)	864 (16.5)	217 (16.2)	322 (17.6)	157 (12.2)	409 (15.4)	97 (17.1)	133 (17.4)
Occupational group								
Managers	196 (5.4)	498 (8.2)	59 (4.0)	127 (6.0)	128 (8.7)	363 (11.7)	9 (1.4)	8 (0.9)
Professionals	291 (8.1)	408 (6.7)	111 (7.5)	123 (5.8)	170 (11.6)	282 (9.1)	10 (1.5)	3 (0.3)
Technicians and associate professionals	587 (16.2)	907 (14.8)	259 (17.4)	312 (14.7)	302 (20.5)	550 (17.8)	26 (3.9)	45 (5.0)
Clerical support workers	694 (19.2)	483 (7.9)	348 (23.4)	220 (10.4)	291 (19.8)	216 (7.0)	55 (8.3)	47 (5.2)
Service and sales workers	829 (22.9)	514 (8.4)	398 (26.8)	256 (12.1)	275 (18.7)	190 (6.1)	156 (23.6)	68 (7.5)
Craft and related trades workers	291 (8.1)	1558 (25.5)	68 (4.6)	384 (18.2)	110 (7.5)	910 (29.4)	113 (17.1)	264 (29.2)
Plant and machine operators and assemblers	364 (10.1)	1192 (19.5)	55 (3.7)	484 (22.9)	117 (7.8)	416 (13.5)	192 (29.1)	292 (32.3)
Elementary occupations	362 (10.0)	543 (8.9)	185 (12.5)	208 (9.8)	79 (5.4)	160 (5.2)	98 (14.9)	175 (19.4)
Occupational social class								
High-skilled clerical	487 (13.5)	906 (14.8)	170 (11.5)	250 (11.8)	298 (20.2)	645 (20.9)	19 (2.9)	11 (1.2)
Low-skilled clerical	2110 (58.3)	1904 (31.2)	1005 (67.7)	788 (37.2)	868 (59.0)	956 (30.9)	237 (35.9)	160 (17.7)
High-skilled manual	293 (8.1)	1565 (25.6)	69 (4.6)	386 (18.3)	110 (7.5)	913 (29.5)	114 (17.3)	266 (29.4)
Low-skilled manual	726 (20.1)	1737 /28.4)	240 (16.2)	692 (32.7)	196 (13.3)	578 (18.7)	290 (43.9)	467 (51.7)
Economic sector								
Industry	1383 (38.2)	2575 (42.0)	413 (27.8)	685 (32.2)	567 (38.5)	1407 (45.4)	403 (60.9)	483 (53.4)
Construction	126 (3.5)	990 (16.2)	66 (4.4)	296 (13.9)	53 (3.6)	558 (18.0)	7 (1.1)	136 (15.0)
Wholesale, retail, food, and accommodation	1429 (39.5)	1332 (21.7)	698 (47.0)	578 (27.2)	526 (35.7)	592 (19.1)	205 (31.0)	162 (17.9)
Transportation	259 (7.1)	868 (14.2)	124 (8.3)	425 (20.0)	104 (7.1)	323 (10.5)	31 (4.7)	120 (13.3)
Financial services	423 (11.7)	363 (5.9)	185 (12.5)	141 (6.7)	223 (15.1)	218 (7.0)	15 (2.3)	4 (0.4)
Total	3620 (37.1)	6128 (62.9)	1480 (41.2)	2115 (58.8)	1470 (32.3)	3081 (67.7)	660 (42.2)	902 (57.8)

EWCS 2010

**Table 2:** Socio-demographic characteristics of subjects included in the analyses, by gender and type of work organization

	All subjects (*N* = 10,374)		Reflexive production (*N* = 2777)		Lean production (*N* = 3619)		Tayloristic production (*N* = 2460)		Traditional organization (*N* = 1516)	
	Women *N* (%)	Men *N* (%)	Women *N* (%)	Men *N* (%)	Women *N* (%)	Men *N* (%)	Women *N* (%)	Men *N* (%)	Women *N* (%)	Men *N* (%)
Age class										
15–24	369 (8.9)	528 (8.5)	105 (8.8)	117 (7.4)	83 (6.8)	168 (7.0)	106 (10.5)	169 (11.7)	75 (10.5)	74 (9.2)
25–34	981 (23.7)	1465 (23.5)	260 (21.7)	336 (21.3)	319 (26.1)	576 (24.0)	228 (22.5)	363 (25.1)	174 (24.4)	190 (23.7)
35–44	1176 (28.4)	1649 (26.5)	352 (29.4)	402 (25.4)	361 (30.0)	642 (26.8)	284 (28.1)	401 (27.7)	177 (24.8)	204 (25.4)
45–54	1065 (25.7)	1548 (24.9)	316 (26.4)	406 (25.7)	309 (25.3)	632 (26.4)	266 (26.3)	316 (21.8)	174 (24.4)	194 (24.2)
55+	543 (13.1)	1024 (16.4)	161 (13.5)	315 (19.9)	145 (11.9)	374 (15.6)	125 (12.4)	197 (13.6)	112 (15.7)	138 (17.2)
Missing	11 (0.3)	15 (0.2)	3 (0.3)	4 (0.3)	4 (0.3)	6 (0.3)	3 (0.3)	2 (0.1)	1 (0.1)	3 (0.4)
European region										
Anglo-Saxon	241 (5.8)	448 (7.2)	58 (4.9)	97 (6.1)	93 (7.6)	204 (8.5)	60 (5.9)	100 (6.9)	30 (4.2)	47 (5.9)
Continental	839 (20.0)	1452 (23.3)	312 (26.1)	424 (26.8)	243 (19.9)	576 (24.0)	152 (15.0)	294 (20.3)	123 (17.3)	158 (19.7)
Eastern	1432 (34.6)	1680 (27.0)	374 (31.2)	401 (25.4)	387 (31.7)	574 (23.9)	390 (38.5)	446 (30.8)	281 (39.4)	259 (32.3)
Scandinavia	335 (8.1)	673 (10.8)	133 (11.1)	227 (14.4)	125 (10.2)	325 (14.0)	42 (4.2)	81 (5.6)	35 (4.9)	40 (5.0)
Southern	743 (17.9)	1012 (16.3)	182 (15.2)	213 (13.5)	210 (17.2)	368 (15.4)	208 (20.6)	278 (19.2)	143 (20.1)	153 (19.1)
Non-EU	562 (13.6)	964 (15.5)	138 (11.5)	218 (13.8)	163 (13.4)	351 (14.6)	160 (15.8)	249 (17.2)	101 (14.2)	146 (18.2)
Occupational group										
Managers	240 (5.8)	431 (6.9)	79 (6.6)	120 (7.6)	135 (11.1)	275 (11.5)	14 (1.4)	25 (1.7)	12 (1.7)	11 (1.4)
Professionals	329 (7.9)	466 (7.5)	116 (9.7)	140 (8.9)	165 (13.5)	267 (11.1)	32 (3.2)	47 (3.3)	16 (2.2)	12 (1.5)
Technicians and associate professionals	455 (11.0)	768 (12.3)	176 (14.7)	250 (15.8)	181 (14.8)	394 (16.4)	73 (7.2)	88 (6.1)	25 (3.5)	36 (4.5)
Clerical support workers	700 (16.9)	461 (7.4)	279 (23.3)	144 (9.1)	254 (20.8)	164 (6.8)	74 (7.3)	84 (5.8)	93 (13.0)	69 (8.6)
Service and sales workers	1282 (30.9)	689 (11.1)	388 (32.4)	219 (13.9)	296 (24.2)	209 (8.7)	273 (27.0)	132 (9.1)	325 (45.6)	129 (16.1)
Craft and related trades workers	353 (8.5)	1649 (26.5)	34 (2.8)	337 (21.3)	78 (6.4)	705 (29.4)	189 (18.7)	475 (32.8)	52 (7.3)	132 (16.4)
Plant and machine operators and assemblers	368 (8.9)	1229 (19.7)	33 (2.8)	264 (16.7)	55 (4.5)	265 (11.1)	219 (21.6)	409 (28.3)	61 (8.6)	291 (36.2)
Elementary occupations	413 (10.0)	528 (8.5)	91 (7.6)	104 (6.6)	57 (4.7)	117 (4.9)	137 (13.5)	185 (12.8)	128 (18.0)	122 (15.2)
Missing	3 (0.1)	8 (0.1)	1 (0.1)	2 (0.1)	0 (0.0)	2 (0.1)	1 (0.1)	3 (0.2)	1 (0.1)	1 (0.1)
Occupational social class										
High-skilled clerical	569 (13.7)	897 (14.4)	195 (16.3)	260 (16.5)	300 (24.6)	542 (22.6)	46 (4.6)	72 (5.0)	28 (3.9)	23 (2.9)
Low-skilled clerical	2437 (58.8)	1918 (30.8)	843 (70.4)	613 (38.8)	731 (59.9)	767 (32.0)	420 (41.5)	304 (21.0)	443 (62.1)	234 (29.1)
High-skilled manual	353 (8.5)	1649 (26.5)	34 (2.8)	337 (21.3)	78 (6.4)	705 (29.4)	189 (18.7)	475 (32.8)	52 (7.3)	132 (16.4)
Low-skilled manual	781 (18.9)	1757 (28.2)	124 (10.4)	368 (23.3)	112 (9.2)	382 (15.9)	356 (35.2)	594 (41.0)	189 (26.5)	413 (51.4)
Missing	3 (0.1)	8 (0.1)	1 (0.1)	2 (0.1)	0 (0.0)	2 (0.1)	1 (0.1)	3 (0.2)	1 (0.1)	1 (0.1)
Economic sector										
Industry	1479 (35.7)	2622 (42.1)	307 (25.7)	571 (36.1)	406 (33.3)	982 (41.0)	550 (54.4)	789 (54.5)	216 (30.3)	280 (34.9)
Construction	124 (3.0)	973 (15.6)	47 (3.9)	199 (12.6)	52 (4.3)	485 (20.2)	13 (1.3)	222 (15.3)	12 (1.7)	67 (8.3)
Wholesale, retail, food, and accommodation	1918 (46.3)	1488 (23.9)	631 (52.7)	463 (29.3)	510 (41.8)	549 (22.9)	359 (35.5)	252 (17.4)	418 (58.6)	224 (27.9)
Transportation	223 (5.4)	782 (12.6)	79 (6.6)	239 (15.1)	63 (5.2)	184 (7.7)	45 (4.5)	151 (10.4)	36 (5.1)	208 (25.9)
Financial services	399 (9.6)	364 (5.8)	133 (11.1)	108 (6.8)	190 (15.6)	198 (8.3)	45 (4.5)	34 (2.4)	31 (4.4)	24 (3.0)
Total	4143 (40.0)	6229 (60.0)	1197 (43.1)	1580 (56.9)	1221 (33.7)	2398 (66.3)	1012 (41.1)	1448 (58.9)	713 (47.0)	803 (53.0)

EWCS 2015

One cluster was composed of 3595 subjects in 2010 and 2777 in 2015, and was characterized by a low degree of horizontal constraints, normative constraints, automatic constraints, repetitiveness, monotony, and quality norms, but lower team work and job rotation (Tables 1 and 2); based on such features, it was interpreted as “reflexive production”.

A second cluster, interpreted as “lean production”, was composed of 4551 subjects in 2010 and 3619 in 2015, and displayed high levels of team work, job rotation, time autonomy, problem-solving, and complexity, although also reporting relatively high levels of several types of constraints indicating exposure to high work pressure, such as presence of norms on quantity and quality of the production performed, tight control by supervisors, and dependency on the work pace of machines and colleagues (Tables 1 and 2).

A third cluster was composed of 1562 subjects in 2010 and 2460 in 2015, and had high levels of horizontal constraints, normative constraints, automatic constraints, repetitiveness, monotony, and low levels of time autonomy, methods autonomy, learning, and problem-solving (Tables 1 and 2); based on these characteristics, this cluster was interpreted as “Tayloristic production”.

The fourth cluster, found only in 2015 data, was also characterized by low levels of time autonomy, methods autonomy, learning, and problem-solving, but repetitiveness and monotony were less prevalent than in Tayloristic production, while levels of horizontal and normative constraints were very low, similar to those of the reflexive production model.

Examining diffusion of the organizational models by European region, in EWCS 2015, lean production was found to be the most common model in the Anglo-Saxon (43.9%) and the Scandinavian regions (41.7%), areas where the traditional model was the least common (10.7% and 10.0%, respectively), while reflexive production was highest in the Scandinavian and Continental regions (36.4% and 34.0%, respectively), and Tayloristic production in the Eastern, Southern, and non-EU regions (26.8%, 27.4% and 26.8%, respectively). For traditional production, the highest prevalence was seen in the Southern and Eastern regions (18.7% and 16.8%, respectively).

In Table 3, prevalences by gender, as well as female-to-male prevalence ratios (PRs) of exposure to psychosocial and ergonomic factors at work are presented for each work organization model and year of the survey. Except for exposure to high job strain in 2015, for all other hazards, the prevalence of exposure was lower among both male and female workers employed in firms adopting the reflexive production model, than among those employed in companies with other organizational models.

**Table 3:** Standardized prevalences^a^, female/male prevalence ratios (PRs) of exposure to unfavourable psychosocial and physical work conditions in 2010 and 2015, by type of work organization, and *p* values for interaction between gender and type of work organization

Work exposure	Reflexive production		Lean production			Tayloristic production			Traditional production		
	%	Female/male PR	%	Female/male PR	*p* value^b^	%	Female/male PR	*p* value^b^	%	Female/male PR	*p* value^b^
High job strain—2010											
Males	22.1	1	24.1	1		77.2	1				
Females	23.4	1.06 (0.94–1.20)	28.5	1.26 (1.14–140)	0.07	86.8	1.16 (1.10–1.22)	0.03			
High job strain—2015											
Males	16.2	1	12.9	1		62.0	1		55.0	1	
Females	18.0	1.14 (0.95–1.37)	17.9	1.43 (1.19–1.73)	0.07	70.8	1.16 (1.10–1.23)	0.51	57.9	1.09 (0.99–1.20)	0.99
High effort–reward imbalance—2010											
Males	23.3	1	31.3	1		52.0	1				
Females	23.6	1.03 (0.91–1.16)	35.9	1.18 (1.08–1.29)	0.10	64.4	1.25 (1.15–1.37)	0.003			
High effort–reward imbalance—2015											
Males	21.9	1	23.7	1		40.4	1		32.1	1	
Females	22.0	1.19 (1.01–1.40)	31.4	1.31 (1.15–1.49)	0.26	50.5	1.24 (1.14–1.36)	0.31	35.6	1.14 (0.97–1.33)	0.89
Tiring or painful postures—2010											
Males	20.4	1	25.0	1		39.8	1				
Females	17.5	1.00 (0.87–1.16)	23.7	1.10 (0.98–1.23)	0.29	47.5	1.26 (1.12–1.42)	0.003			
Tiring or painful postures—2015											
Males	17.8	1	21.2	1		31.9	1		21.3	1	
Females	19.2	1.26 (1.04–1.53)	26.2	1.40 (1.21–1.62)	0.12	38.4	1.29 (1.16–1.43)	0.34	23.3	1.14 (0.95–1.38)	0.99
Carrying or moving heavy loads—2010											
Males	15.1	1	19.4	1		31.2	1				
Females	6.8	0.48 (0.38–0.59)	10.7	0.62 (0.53–0.74)	0.09	14.1	0.49 (0.39–0.62)	0.99			
Carrying or moving heavy loads—2015											
Males	14.4	1	18.6	1		27.9	1		19.9	1	
Females	8.2	0.73 (0.57–0.95)	15.5	0.99 (0.82–1.19)	0.06	17.8	0.62 (0.53–0.73)	0.55	9.1	0.44 (0.33–0.59)	0.05
Repetitive hand or arm movements—2010											
Males	39.5	1	45.3	1		66.6	1				
Females	44.2	1.23 (1.14–1.34)	53.2	1.25 (1.17–1.33)	0.50	81.1	1.24 (1.16–1.33)	0.23			
Repetitive hand or arm movements—2015											
Males	34.4	1	42.2	1		55.9	1		37.4	1	
Females	46.9	1.56 (1.40–1.73)	58.8	1.47 (1.36–1.59)	0.99	66.3	1.21 (1.14–1.29)	0.03	50.4	1.39 (1.24–1.57)	0.21

Poisson robust regression models adjusted for age, European region, occupational social class, and economic sector

^a^All prevalences standardized by age class, European region, occupational social class, and economic sector

^b^*p* value for interaction between gender and type of work organization on the PR of exposure to unfavourable work factors, with reflexive production as the reference work organizational model

In both surveys, women had a higher likelihood of exposure than males to unfavourable work factors in all types of work organization, except for carrying or moving heavy loads. Only for job strain and moving/carrying heavy loads results consistently showed across surveys an exposure profile more favourable to women in the reflexive production than in the lean and Tayloristic production.

In detail, for high job strain in 2010, the female/male PRs were significantly higher in the lean production (PR = 1.26, 1.14–1.40) and in the Tayloristic production (PR = 1.16, 1.10–1.22), compared to reflexive production (1.06–0.94–1.20) (*p* value for interaction = 0.07 and 0.03, respectively), whereas in 2015, only the PR for the lean production was significantly higher (PR = 1.43, 1.19–1.73 vs. PR = 1.14, 0.95–1.3; *p* value for interaction = 0.07). For ERI, in 2010, the female/male PR of the reflexive production (PR = 1.03, 0.91–1.6) was significantly lower than that of the other two models (lean: PR = 1.18, 1.08–1.29; Tayloristic: PR = 1.25, 1.15–1.37) (*p* value for interaction = 0.10 and 0.003, respectively), whereas in 2015, no significant differences were observed among the four models.

Regarding ergonomic hazards, in 2010, the female/male PR for awkward postures observed in reflexive production was significantly lower than that in Tayloristic production (PR = 1.00, 0.87–1.16 vs. PR = 1.24, 1.14–1.36; *p* value for interaction = 0.003), whereas no significant difference was observed in 2015. Also, in both surveys, a significantly lower female/male PR for carrying/moving heavy loads was found in reflexive production compared to lean production (PR = 0.48, 0.38–0.59 vs. PR = 0.62, 0.53–0.74 in 2010; PR = 0.73, 0.57–0.95 vs. PR = 0.99, 0.82–1.19 in 2015), although in 2015, it was higher than that observed in the traditional model (PR = 0.44, 0.33–0.59). Finally, no significant differences were observed for repetitive movements across the three models in 2010, whereas in 2015, gender PR were highest in reflexive production, and significantly higher than in Tayloristic production (PR = 1.56, 1.40–1.73 vs. PR = 1.21, 1.14–1.29; *p *value for interaction = 0.03).

Regarding health, both low mental well-being and musculoskeletal pain were more prevalent in females than males (Table 4). For low mental well-being, in 2010, gender PRs did not show any significant difference across the different organizational models, whereas in 2015, the PR was highest in reflexive production (PR = 1.49, 1.22–1.81), and significantly higher than that in traditional production (PR = 1.13, 0.89–1.42). For musculoskeletal pain, no significant differences in gender PRs between the different organizational models were observed in the 2015 survey, whereas in 2010 the female/male PR of backache in reflexive production was significantly lower than that in Tayloristic production (PR = 1.06, 0.98–1.14 vs. PR = 1.18, 1.08–1.30; *p* value for interaction = 0.03), while that of upper limb musculoskeletal pain was significantly lower than that in lean production (PR = 1.16, 1.06–1.25 vs. PR = 1.24, 1.16–1.33; *p* value for interaction = 0.05).

**Table 4:** Standardized prevalences^a^, female/male prevalence ratios (PRs) of mental and physical health conditions in 2010 and 2015, by type of work organization, and *p* values for interaction between gender and type of work organization

Health conditions	Reflexive production		Lean production			Tayloristic production			Traditional production		
	%	Female/male PR (95% CI)	%	Female/male PR (95% CI)	*p* value^b^	%	Female/male PR (95% CI)	*p* value^b^	%	Female/male PR (95% CI)	*p* value^b^
Low mental well-being—2010											
Males	18.1	1	17.9	1		29.6	1				
Females	22.5	1.25 (1.10–1.42)	23.2	1.37 (1.19–1.58)	0.34	36.1	1.18 (1.02–1.38)	0.16			
Low mental well-being—2015											
Males	13.6	1	12.5	1		20.8	1		18.1	1	
Females	15.1	1.49 (1.22–1.81)	17.6	1.39 (1.16–1.67)	0.13	24.5	1.23 (1.06–1.44)	0.12	18.2	1.13 (0.89–1.42)	0.03
Back MSD—2010											
Males	44.4	1	46.2	1		50.7	1				
Females	44.6	1.06 (0.98–1.14)	48.8	1.09 (1.02–1.16)	0.42	59.5	1.18 (1.08–1.30)	0.03			
Back MSD—2015											
Males	41.5	1	45.3	1		50.5	1		37.4	1	
Females	46.1	1.12 (1.02–1.23)	48.2	1.09 (1.00–1.18)	0.72	56.5	1.12 (1.04–1.21)	0.64	41.6	1.22 (1.07–1.38)	0.49
Upper limb MSD—2010											
Males	39.4	1	42.9	1		49.0	1				
Females	42.3	1.16 (1.06–1.25)	51.2	1.24 (1.16–1.33)	0.05	57.9	1.19 (1.08–1.32)	0.18			
Upper limb MSD—2015											
Males	38.7	1	42.8	1		49.0	1		37.1	1	
Females	44.5	1.28 (1.16–1.41)	54.1	1.33 (1.22–1.44)	0.51	59.5	1.15 (1.07–1.25)	0.30	40.5	1.18 (1.03–1.36)	0.42

Poisson robust models adjusted for age class, European region, occupational social class, and economic sector

^a^All prevalences standardized by age class, European region, occupational social class, and economic sector

^b^*p* value for interaction between gender and type of work organization on the PR of exposure to unfavourable work factors, with reflexive production as the reference work organizational model

The comparison between 2010 and 2015 overall samples showed similar prevalences of exposure to work factors and of health outcomes in both men and women, except for low mental well-being, whose prevalence decreased in 2015 by almost 5% in men and 7% in women (Table 5). No significant difference in gender PRs for any work factor or health condition was found between the two surveys.

**Table 5:** Standardized prevalences^a^ and prevalence ratios of work characteristics and health conditions by gender and year of the survey

Work characteristics	EWCS 2010			EWCS 2015			Diff. EWCS 2010–2015
	Men	Women	PR women/men	Men	Women	PR women/men	*p* value^b^
High job strain	30.8	38.5	1.33 (1.26–1.42)	29.7	38.6	1.34 (1.26–1.42)	0.86
High effort-reward imbalance	31.2	37.6	1.23 (1.16–1.31)	27.4	33.5	1.29 (1.21–1.37)	0.28
Tiring or painful postures	23.5	28.0	1.31 (1.21–1.41)	21.7	25.7	1.32 (1.22–1.42)	0.89
Carrying or moving heavy loads	18.1	10.9	0.64 (0.57–0.71)	19.2	12.2	0.70 (0.63–0.77)	0.24
Repetitive hand or arm movements	44.1	56.9	1.38 (1.32–1.44)	42.1	56.5	1.41 (1.35–1.47)	0.49
Low mental well-being	19.7	25.9	1.32 (1.22–1.43)	15.0	19.1	1.36 (1.24–1.49)	0.63
Back MSD	45.0	50.1	1.16 (1.11–1.22)	44.1	47.4	1.12 (1.07–1.17)	0.29
Upper limb MSD	41.1	50.6	1.28 (1.22–1.34)	42.0	49.6	1.24 (1.19–1.30)	0.34

Gender prevalence ratios estimated through Poisson robust regression models, adjusted for age, European region, occupational social class, and economic sector

^a^All prevalences standardized by age class, European region, occupational social class, and economic sector

^b^Differences between gender prevalence ratios by year of the survey (2010, 2015) were tested assessing heterogeneity of the PRs through fixed-effect meta-analysis

In general, female/male PRs of exposure to work factors and of health outcomes were stronger in Continental, Eastern, Scandinavian, and non-EU countries, while lower in Anglo-Saxon and Southern countries, with significant differences among regions for all the variables examined, except for low mental health and musculoskeletal pain in the upper limb (Supplementary Table 3). In contrast, Gender PRs did not differ significantly between EU and non-EU countries, except for carrying/moving heavy loads, to which women were less exposed in non-EU countries (data not shown).

## Discussion

As expected, in European workplaces, women were more likely than men to be exposed to the psychosocial and physical hazards examined, except for carrying or moving heavy loads, and were more likely to report low mental well-being and musculoskeletal pain.

Regarding differences in exposure to work hazards between men and women across the different organizational models, we found in both 2010 and 2015 surveys that the female/male prevalence ratio of exposure to work stress, defined according to the job demand-control model (Karasek 1979), was significantly lower in workplaces characterized by the reflexive production model, compared to those employing the lean production. In contrast, results for ERI were discordant between the two surveys, with significantly or marginally significantly higher gender PRs in lean and Tayloristic production, compared to reflexive production, and no differences in 2015. With respect to ergonomic hazards, smaller differences were observed by gender, although in both surveys, the female/male PRs of exposure to carrying/moving heavy loads were significantly lower in reflexive production than in lean production. Concerning health, in 2010, gender differences in musculoskeletal pain were more favourable to women in reflexive production, compared to the other two models, even if with some differences for back and upper limb pain, whereas no differences were present in 2015. For low mental well-being, no differences in the gender PRs were present across the three models in 2010, although in 2015, the PR in reflexive production was higher than that of all other models, and significantly different from that of traditional production.

In summary, we found consistent evidence across the two surveys only for a lower female/male ratio of exposure to job strain and to heavy loads in reflexive production, compared to lean production. It seems unlikely that differences in the results observed between the two surveys could derive from actual changes in working conditions occurred during this period, given the relatively short time elapsed, but rather to chance, also considering that interactions between gender and organizational model in 2010 for other work exposures and for health conditions were mostly of borderline significance. However, gender PRs in the overall sample were quite consistent across surveys, suggesting that differences in the number of organizational models identified in 2010 and 2015 may have contributed to limit comparability of the results by type of organization between the two surveys.

A possible interpretation of the gender differences in exposure to job strain observed between reflexive and lean production is that the reflexive production organization is less gendered. Indeed, some scholars have mentioned the learning organization as a type of organization more conducive to women, as it is able to construct an environment of learning, which gives value to the individuals and their competences and experiences (Cram et al. 2016; Johansson and Abrahamsson 2018; Luciano 2008; Raaijmakers et al. 2018). This type of organizational context would be more open to value diversity and therefore to women’ culture and attitudes. However, learning new things and problem-solving in our analysis were both more diffuse in the lean production model. Concerning workplace learning, it is possible that the employees do not recognize ways of more informal learning occurring in their daily work (Evans 2002). The framing of the question in the questionnaire—“learning new things”—might be interpreted by the responders as referring to explicit learning activities. In the lean production organization, learning could be more evident, thanks to the constant request and effort to improve the work. Workplaces organized in accordance with the reflexive production model may be characterized by a subtle way of learning (Billett 2004), less recognizable by the workers but however present, linked to a more interdisciplinary, integrated and interactive work (Lundvall et al. 2011). These features may create a workplace environment accustomed to diversity and different points of view, and therefore more favourable to female employees. According to some authors, learning in this type of work organization is an experienced-based learning, emerging from “doing, using and interacting”, characterized by strong elements of tacit knowledge. This happens thanks to multidisciplinary workgroups, integration of functions, and closer interaction with customers (Lundvall et al. 2011).

Looking closer at the differences between reflexive and lean production highlights some more aspects relevant to interpret the gender gap in exposure to job strain. The only features which seem to explain the lower female/male PRs of exposure to job strain in the reflexive production model are related to the higher monotony, higher repetitiveness, and higher exposure to different types of constraints, all characteristics more diffused in lean production. In particular, it seems likely that more strict quantitative production and quality norms in lean production, together with direct control by supervisors, would increase work intensity and effort requested to workers, an effect already pointed out in the literature critical toward the lean production. Different studies on the subject found that this type of work organization brings about intensified work pace and demands, and job strain (Arezes et al. 2015; Bouville and Alis 2014; Landsbergis et al. 1999; Oudhuis and Tengblad 2020; Stewart et al. 2016). Yet, a growing literature points out the role of management practices in the way lean production is implemented, which may to limit work intensification and workers’ strain (Bocquet et al. 2019; Koukoulaki 2014; Longoni et al. 2013; Neirotti 2020; Stimec and Grima 2019), although this might not work in certain sectors (Ogbonnaya et al. 2017). Such a work intensification could be experienced especially by female workers, who are more segregated in lower hierarchical positions, and this in turn would raise their exposure to work stress and ergonomic hazards. Some scholars have already noted that the introduction of organizational forms of lean production does not favour women, nor it affects gender segregation (Abrahamsson 2014; Losonci et al. 2011; Zanoni 2011). Although diverse applications of lean production exist, depending on cultural and institutional contexts, with different mixes of elements of the ideal-type of lean production, in general, the involvement of workers appears as problematic; the studies of Babson (1995), Rinehart and colleagues (1997), and Appelbaum and Batt (1993) have found that work in lean production is actually organized in a way that often is accompanied by job security reduction, lack of promotion, and weak representation of workers’ interests. This problematic aspect of the lean production model seems to persist: recent works still discuss the need for improvement of the workers’ participative processes, their autonomy, and their learning (Lantz et al. 2015; Stimec and Grima 2019), which are lacking where the lean production principles are misapplied (Neirotti 2020), a situation which appears to be frequent (Arezes et al. 2015). The finding in our study that both men and women employed in companies belonging to reflexive production reported a lower prevalence of exposure to psychosocial and physical hazards, compared to the other types of work organization, gives support to the theory that such a model is characterized by working conditions more acceptable to workers of both genders.

The lesser job constraints experienced in the reflexive production model and the much lower frequency of teamwork could be the main features which favour the lower female/male prevalence ratio of work stress in this organizational model. One interpretation could be that these features allow women to enjoy more freedom and higher control on their jobs, and that they can reduce the pressure of a male-gendered organization with its dominant male-culture and practices. This would decrease women’s exposure to psychological demands and increase job control, with a consequent reduction in their level of job strain, compared to other organizational models. In particular, the lower frequency of teamwork could give women even more chance to avoid stress and lack of recognition. Williams and colleagues (2012) note that teamwork, very often supervised by male staff, tends to obscure individual contribution and put more stress on women to promote themselves and to receive credit from their supervisors and peers. As much literature has demonstrated, women find difficulties in receiving non-paternalist support in their carrier and “are given disproportionately less credit than men for the success they achieve when they work on teams in male-dominated environments …” (ibidem, 557). It is possible to speculate that, given that too often the organizational processes are still governed by gender prejudices and stereotypes, in flatter organization teamwork can play in women’s disfavour. Feminist theories argue that women need freedom in a male-dominate environment to express themselves in autonomy with respect to gender roles socially constructed (Bertell 2016; Dini and Tarantino 2014; Youngblood Jackson 2013).

Among strengths of this study, it employed a large representative sample of the European working population, which on one hand provided the study with substantial statistical power, and on the other hand permits to generalize the findings to private employees living in the countries included in the survey. Although information on the work environment was self-reported, questions used in EWCS surveys to assess exposure to psychosocial and physical factors at work have been extensively validated (Wikman 1991), and in the last decades, many studies have used EWCS data for conducting occupational and social epidemiological research.

Nonetheless, the self-reported nature of information on working conditions and health does not allow to exclude that the higher prevalences observed among women may be attributable to an overestimation of exposure and health conditions in women, compared to men. However, as commented in the introduction, several studies have found, consistently with our results, higher exposure to most physical and psychosocial factors at work among women. Also for mental and physical health, women show in the literature a consistently higher likelihood of depressive symptoms (Salk et al. 2017) and musculoskeletal pain (Andorsen et al. 2017; de Zwart et al. 2001), compared to men. Another limitation is the lack of information on domestic workload, in terms of household duties and child care, as the greater family burden sustained by women (Anxo et al. 2011) may have concurred in determining the higher prevalences observed among women than men, in particular for health outcomes (Bilodeau et al. 2020; Beauregard et al. 2018).

Regarding the cross-sectional design of the study, as we compared exposure to work factors and health outcomes between men and women, overall and by organizational model, without investigating associations between exposure and health outcomes, the lack of temporality characteristic of this type of study is not expected to have biased in an important way the results. However, it seems difficult to exclude that women and men in the samples analyzed have been subjected to a different degree of selection in and out of the workforce.

In conclusion, a few gender differences were consistently observed in exposure to adverse physical and psychosocial factors at work among the different organizational models examined. However, the lower female/male ratio of exposure to job strain observed in both surveys in reflexive production, compared to lean production, seems to indicate that this work organizational model may be favourable to women, possibly because of a more limited amount of team work and a lower degree of hierarchical constraints characteristic of this type of organization.

This study suggests that the adoption of a work organization point of view allows to elaborate further on gender differences in well-being and health in workplaces, compared to the studies considering only type of industry, job title, tasks, and activities. The work organization concept permits to make sense of bundles of working conditions and open the discourse up to include the gendered dimension of work organization. Compared to the gender-based segregation approach, it allows to consider that even in a male-dominated work environment, the work organization could present features which grant more freedom to women. This may be the case of the reflexive production, where teamwork is less adopted, although, based on our results, we can only speculate that less teamwork plays in favour of women’s health. Our results indicate that female well-being in the workplace needs to be investigated further, drawing attention to specific gender issues. The literature on gendered organization theory seems one of the perspectives more promising in providing advancement to the field.

## Supplementary Information

Below is the link to the electronic supplementary material.

Supplementary file1 (DOCX 16 KB)

Supplementary file2 (DOCX 19 KB)

Supplementary file3 (DOCX 17 KB)
