# Supplementary material for: Impact of different work organizational models on gender differences in exposure to psychosocial and ergonomic hazards at work and in mental and physical health
Source: Int Arch Occup Environ Health. 2021 May 29;94(8):1889–904. doi: 10.1007/s00420-021-01720-z (PMC8490231; doi:10.1007/s00420-021-01720-z)
Supplement: Supplementary file 1 — Supplementary file1 (DOCX 16 KB) [file 420_2021_1720_MOESM1_ESM.docx]

**Supplementary Table 1. Prevalences of work characteristics by type of work organization and gender, standardized by age class, European region, occupational social class, and economic sector (6,128 men and 3,621 women). EWCS 2010.**

|  | | | |  | | | |
| --- | --- | --- | --- | --- | --- | --- | --- |
|  | **DISCRETIONARY LEARNING** | | **LEAN PRODUCTION** | | | **TAYLORISTIC PRODUCTION** | |
| **Work characteristics** | **Men** | **Women** | **Men** | | **Women** | **Men** | **Women** |
|  | **%** | **%** | **%** | | **%** | **%** | **%** |
| team work | 46.3 | 42.1 | 80.6 | | 82.7 | 68.0 | 64.7 |
| job rotation | 31.8 | 31.2 | 64.4 | | 66.4 | 54.0 | 52.6 |
| time autonomy | 88.4 | 86.8 | 91.2 | | 91.7 | 31.4 | 22.3 |
| methods autonomy | 61.2 | 63.1 | 72.5 | | 70.3 | 6.0 | 9.1 |
| horizontal constraints | 30.4 | 27.3 | 69.6 | | 70.8 | 72.4 | 65.4 |
| normative constraints | 23.5 | 17.5 | 63.4 | | 64.2 | 68.6 | 64.8 |
| automatic constraints | 10.9 | 4.8 | 35.7 | | 32.6 | 67.5 | 56.7 |
| hierarchical constraints | 28.1 | 28.9 | 56.7 | | 59.4 | 71.4 | 69.6 |
| repetitiveness | 17.8 | 22.1 | 29.6 | | 35.4 | 44.2 | 60.0 |
| monotony | 36.6 | 41.2 | 47.2 | | 55.8 | 79.7 | 80.9 |
| complexity | 43.5 | 38.7 | 84.6 | | 79.8 | 30.7 | 23.6 |
| learning | 54.3 | 53.6 | 91.8 | | 92.1 | 28.5 | 29.2 |
| problem solving | 75.8 | 69.3 | 96.0 | | 93.7 | 41.4 | 36.7 |
| individual quality assessment | 58.0 | 59.3 | 91.7 | | 93.7 | 47.6 | 51.5 |
| quality norms | 60.1 | 60.6 | 94.5 | | 93.5 | 82.3 | 83.2 |
