# Supplementary material for: Impact of different work organizational models on gender differences in exposure to psychosocial and ergonomic hazards at work and in mental and physical health
Source: Int Arch Occup Environ Health. 2021 May 29;94(8):1889–904. doi: 10.1007/s00420-021-01720-z (PMC8490231; doi:10.1007/s00420-021-01720-z)
Supplement: Supplementary file 2 — Supplementary file2 (DOCX 19 KB) [file 420_2021_1720_MOESM2_ESM.docx]

**Supplementary Table 2. Prevalences of work characteristics by type of work organization and gender, standardized by age class, European region, occupational social class, and economic sector (6,229 men and 4,143 women). EWCS 2015.**

|  | | |  | | | |  | | |
| --- | --- | --- | --- | --- | --- | --- | --- | --- | --- |
|  | **REFLEXIVE PRODUCTION** | | **LEAN PRODUCTION** | | **TAYLORISTIC PRODUCTION** | | **TRADITIONAL PRODUCTION** | | |
|  | **Men** | **Women** | **Men** | **Women** | **Men** | **Women** | **Men** | **Women** |  |
| **Work characteristics** | **(%)** | **(%)** | **(%)** | **(%)** | **(%)** | **(%)** | **(%)** | **(%)** |  |
| team work | 44.3 | 41.2 | 83.7 | 85.0 | 68.2 | 69.1 | 26.9 | 31.0 |  |
| job rotation | 31.9 | 30.7 | 70.9 | 75.1 | 66.2 | 68.0 | 25.1 | 31.1 |  |
| time autonomy | 87.9 | 89.4 | 95.3 | 95.4 | 44.4 | 40.6 | 45.2 | 42.6 |  |
| methods autonomy | 74.9 | 71.1 | 87.6 | 87.4 | 23.7 | 20.9 | 19.8 | 20.8 |  |
| horizontal constraints | 26.0 | 23.9 | 69.2 | 67.3 | 78.9 | 80.4 | 29.3 | 29.7 |  |
| normative constraints | 25.8 | 24.6 | 63.1 | 61.1 | 76.0 | 78.0 | 28.7 | 27.9 |  |
| automatic constraints | 8.2 | 7.1 | 31.4 | 29.3 | 62.3 | 57.5 | 26.3 | 22.2 |  |
| hierarchical constraints | 28.3 | 25.9 | 50.9 | 53.0 | 75.1 | 77.2 | 41.3 | 38.0 |  |
| repetitiveness | 13.7 | 19.1 | 23.9 | 33.3 | 45.2 | 53.0 | 18.7 | 30.6 |  |
| monotony | 32.4 | 40.5 | 46.6 | 54.3 | 76.6 | 80.6 | 55.9 | 58.3 |  |
| complexity | 54.1 | 44.6 | 90.4 | 86.2 | 58.2 | 50.9 | 15.1 | 12.2 |  |
| learning | 66.7 | 69.5 | 95.1 | 97.1 | 62.3 | 66.3 | 20.1 | 18.4 |  |
| problem solving | 89.1 | 84.7 | 98.7 | 97.5 | 78.2 | 71.0 | 41.2 | 32.1 |  |
| individual quality assessment | 68.7 | 71.3 | 93.7 | 94.9 | 76.1 | 75.9 | 27.0 | 29.6 |  |
| quality norms | 65.9 | 65.6 | 94.3 | 93.0 | 92.1 | 92.9 | 47.5 | 47.9 |  |
