# Supplementary material for: Impact of different work organizational models on gender differences in exposure to psychosocial and ergonomic hazards at work and in mental and physical health
Source: Int Arch Occup Environ Health. 2021 May 29;94(8):1889–904. doi: 10.1007/s00420-021-01720-z (PMC8490231; doi:10.1007/s00420-021-01720-z)
Supplement: Supplementary file 3 — Supplementary file3 (DOCX 17 KB) [file 420_2021_1720_MOESM3_ESM.docx]

**Supplementary Table 3. Female/male Prevalence Ratios of work characteristics and health conditions by European region in EWCS 2015.^a^**

|  | **Anglo-saxon** | **Continental** | **Eastern** | **Scandinavian** | **Southern** | **Non-EU** |  |
| --- | --- | --- | --- | --- | --- | --- | --- |
| **Outcome** | **PR (95% CI)** | **PR (95% CI)** | **PR (95% CI)** | **PR (95% CI)** | **PR (95% CI)** | **PR (95% CI)** | **p-value ^b^** |
| High job strain | 1.06 (0.81-1.40) | 1.45 (1.28-1.65) | 1.33 (1.20-1.47) | 1.52 (1.18-1.96) | 1.15 (1.02-1.29) | 1.43 (1.24-1.65) | 0.03 |
| High effort-reward imbalance | 0.78 (0.57-1.08) | 1.29 (1.13-1.49) | 1.48 (1.32-1.67) | 1.58 (1.22-2.03) | 1.02 (0.89-1.16) | 1.31 (1.11-1.54) | <0.001 |
| Tiring or painful postures | 1.31 (0.83-2.06) | 1.34 (1.11-1.61) | 1.39 (1.23-1.58) | 1.90 (1.31-2.77) | 1.04 (0.91-1.20) | 1.35 (1.13-1.60) | 0.01 |
| Carrying or moving heavy loads | 0.87 (0.56-1.35) | 0.77 (0.61-0.97) | 0.68 (0.57-0.82) | 1.17 (0.77-1.79) | 0.65 (0.52-0.80) | 0.48 (0.35-0.65) | 0.035 |
| Repetitive hand or arm movements | 1.27 (1.04-1.54) | 1.55 (1.40-1.72) | 1.47 (1.37-1.58) | 1.36 (1.17-1.57) | 1.20 (1.10-1.31) | 1.36 (1.22-1.52) | 0.002 |
| Low mental well-being | 0.97 (0.68-1.38) | 1.48 (1.22-1.79) | 1.38 (1.16-1.54) | 1.45 (1.03-2.04) | 1.18 (0.92-1.52) | 1.27 (1.02-1.60) | 0.25 |
| Back MSD | 0.96 (0.74-1.25) | 1.04 (0.94-1.14) | 1.18 (1.08-1.27) | 0.95 (0.80-1.12) | 1.17 (1.05-1.30) | 1.21 (1.08-1.36) | 0.035 |
| Upper limb MSD | 1.09 (0.86-1.38) | 1.30 (1.18-1.44) | 1.28 (1.18-1.40) | 1.19 (1.04-1.35) | 1.22 (1.10-1.37) | 1.17 (1.04-1.31) | 0.54 |

^a^ Gender Prevalence Ratios estimated through Poisson robust regression models, adjusted for age, occupational social class, and economic sector

^b^ Differences between gender Prevalence Ratios by European region were tested assessing heterogeneity of the PRs through random-effect meta-analysis
